# Rectal irrigation as rescue therapy for refractory and severe hemorrhagic radiation proctitis: A case report

**DOI:** 10.1002/ccr3.4985

**Published:** 2021-10-21

**Authors:** Sukit Pattarajierapan, Napapat Amornwichet, Supakij Khomvilai

**Affiliations:** ^1^ Surgical Endoscopy Colorectal Division, Department of Surgery Faculty of Medicine, Chulalongkorn University Bangkok Thailand; ^2^ Division of Radiation Oncology, Department of Radiology Faculty of Medicine, Chulalongkorn University Bangkok Thailand

**Keywords:** argon plasma coagulation, diverting colostomy, gastrointestinal hemorrhage, radiation proctitis, rectal irrigation

## Abstract

Rectal irrigation may be considered in refractory and severe hematochezia from chronic radiation proctitis before performing other invasive treatments. It prevents superimposed infection and effectively reduces bleeding.

## Graphical abstract

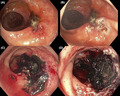

## 1 INTRODUCTION

Hemorrhagic chronic radiation proctitis (CRP) refractory to endoscopic therapy is rare. Because of its high morbidity and mortality rates, proctectomy is considered as the last resort. We report the successful treatment of severe hematochezia refractory to endoscopic therapy and diverting colostomy via rectal irrigation in a patient with CRP.

Radiotherapy (RT) is often used in the management of pelvic cancers, including urologic, gynecologic, and gastrointestinal malignancies. However, the possibility of radiation toxicity has limited its utility.

Chronic radiation proctitis (CRP) has been reported in 5%–20% of patients following RT,^1^, ^2^ often presenting with hematochezia. RT‐induced endarteritis obliterans causes intimal fibrosis and fibrin thrombi formation of the small arterioles leading to ischemia and telangiectasia that can cause bleeding.^3^

Argon plasma coagulation (APC) is an effective endoscopic therapy for most patients with hemorrhagic CRP.^4^ However, to the best of our knowledge, the efficacy of APC in patients with CRP who received high‐dose RT remains unknown. Moreover, in these patients, APC may aggravate bleeding due to the impaired recovery of the rectum. Herein, we report the successful treatment of severe hematochezia refractory to endoscopic therapy and diverting colostomy via rectal irrigation in a patient with CRP.

## 2 CASE

A 48‐year‐old woman with stage IIIC2 cervical cancer presented with severe vaginal bleeding. To control the bleeding, she underwent emergency external beam RT and brachytherapy. She was then treated with curative high‐dose volumetric modulated arc RT and three‐dimensional brachytherapy. The total radiation dose in the anterior region of the mid‐rectum reached 150 Gy. Due to the presence of residual disease, she received six additional cycles of chemotherapy with carboplatin and paclitaxel. Six months after RT completion, she developed severe hematochezia with associated reduction of hemoglobin levels (1.7 g/dl), which prompted transfusion with packed red cells.

**FIGURE 1:**
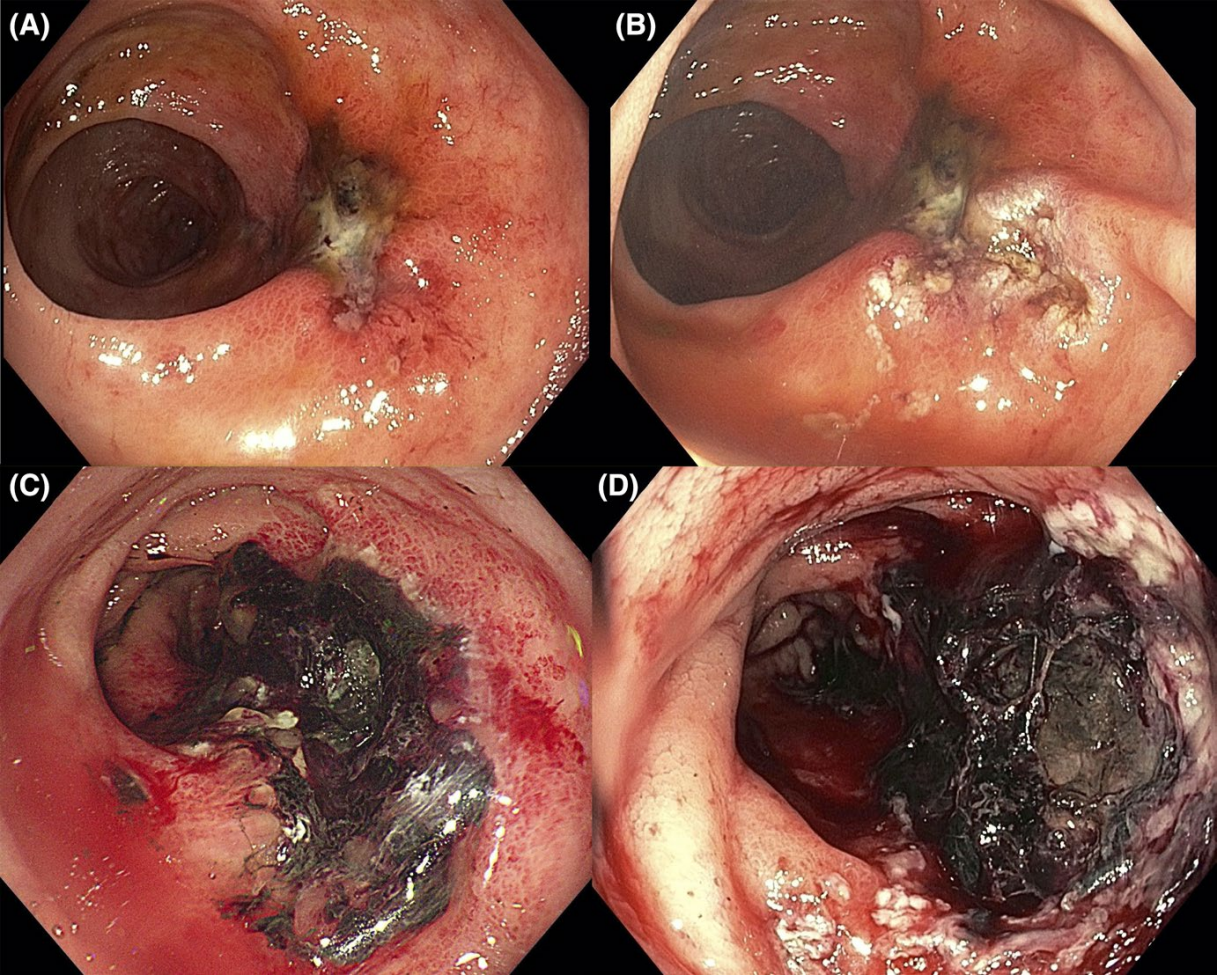
Endoscopic images: (A) Colonoscopy shows ulceration with minimal bleeding, consistent with radiation proctitis. (B) Argon plasma coagulation immediately stops the rectal ulcer bleeding. (C) Colonoscopy 1 month after argon plasma coagulation demonstrates worsening ulceration covered with necrotic tissue. (D) Colonoscopy 1 month after diverting colostomy reveals extensive necrotic ulcers with active bleeding

Emergency colonoscopy revealed ulceration with minimal bleeding and rectosigmoid stricture (Figure 1A), consistent with CRP. APC with a power setting of 40 W and a gas flow of 0.8 L/min was performed to stop the bleeding (Figure 1B). Two days after the procedure, the patient was discharged with an uneventful course.

However, after 1 month, her hematochezia persisted. Colonoscopy demonstrated worsening ulceration covered with necrotic tissue (Figure 1C). Re‐treatment with APC did not improve her extensive hemorrhagic CRP; therefore, we performed diverting colostomy to slow down the bleeding. However, she presented with episodic hematochezia and reduced hemoglobin levels (5 g/dl) after 1 month, which prompted a re‐evaluation. Colonoscopy showed extensive necrotic ulcers with active bleeding (Figure 1D). During this time, the bleeding was refractory to APC.

**FIGURE 2:**
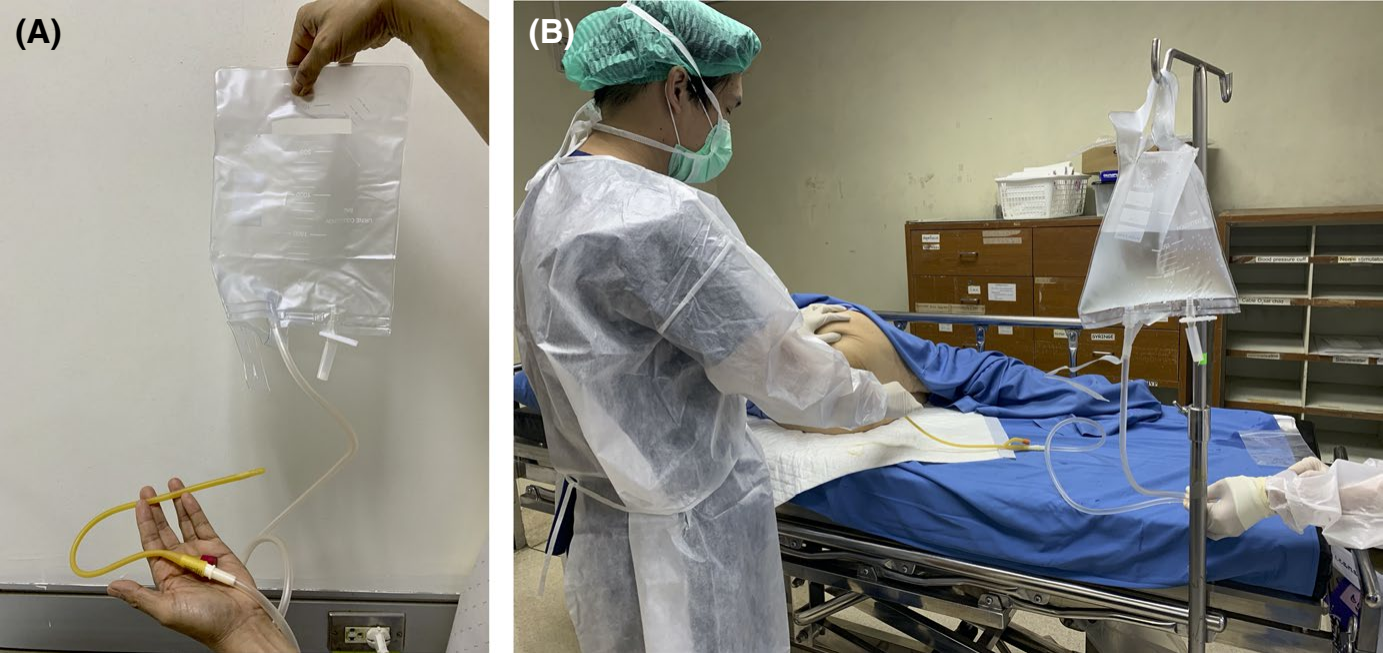
Rectal irrigation in severe hemorrhagic radiation proctitis: (A) The rectal irrigating device that we designed using a Foley catheter No. 16 and a urinary drainage bag. (B) The image demonstrates rectal irrigation with tap water (1 L), which we performed twice a day

**FIGURE 3:**
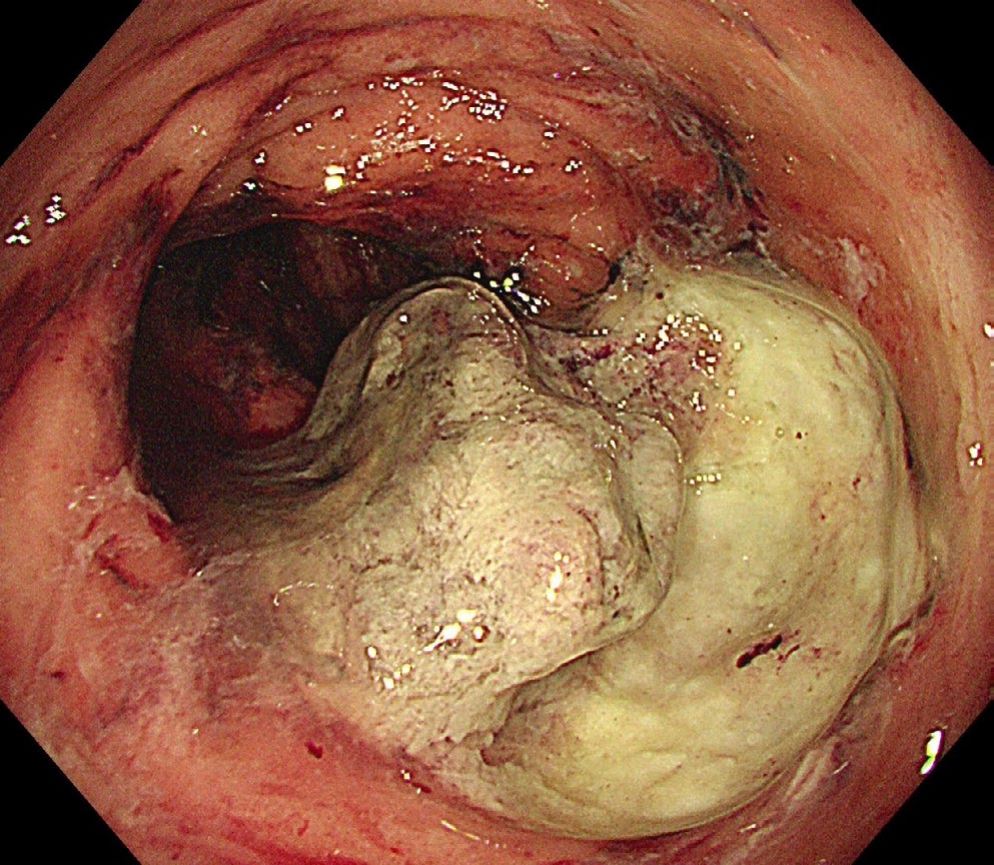
Colonoscopy performed 2 months after self‐administered rectal irrigation shows an organizing rectal ulcer with no active bleeding

Angiography with embolization carries a high risk of rectal necrosis. Moreover, proctectomy in patients who underwent high‐dose RT is associated with high morbidity and mortality; consequently, we performed non‐invasive methods first. We attempted rectal irrigation with tap water (1 L) twice a day using a rectal irrigating device that we designed (Figure 2). Oral ciprofloxacin (500 mg twice daily) and metronidazole (500 mg three times daily) were also prescribed. In theory, rectal irrigation can reduce bleeding and prevent superimposed infection at the same time. After a few days, her bleeding was finally controlled with an associated elevation of the hemoglobin level (10 g/dl). After discharge, she was advised to continue self‐administered rectal irrigation daily. Follow‐up examination revealed persistent control of bleeding with no change in the hemoglobin level. Colonoscopy performed after 2 months revealed an organizing rectal ulcer with no active bleeding (Figure 3).

## 3 DISCUSSION

Patients with CRP usually present with hematochezia. Among the various medical and endoscopic therapies utilized to achieve hemostasis, APC is a widely used non‐contact thermal endoscopic modality with limited depth of penetration. APC has been considered as the treatment of choice for CRP.^1^ A pooled analysis revealed that APC had a high overall success rate of 87%, with a mean or median number of treatment sessions ranging from 1 to 3.7.^5^ However, in patients who received high‐dose RT, the impaired recovery of the rectum after APC might worsen the bleeding. In their study, Canard et al.^6^ found that three (10%) patients had severe complications after APC (i.e., extensive rectal necrosis, perforation, and severe bleeding). While they did not report the radiation dose or type of treatment used, they found that a power setting >45 W was associated with severe complications.^6^

In severe CRP, surgery is considered the last option. It ranges from diverting colostomy to proctectomy with or without an anastomosis.^7^ Diverting the fecal stream via a colostomy, or an ileostomy, can improve the radiation‐induced symptoms, including bleeding. Yaun et al.^8^ showed that diverting colostomy obtained a higher bleeding remission than the conservative treatment (94% vs. 12%) in severe hemorrhagic CRP. In our patient, diverting colostomy failed to slow down the bleeding from CRP. Generally, the only option for this patient would be proctectomy. However, proctectomy is associated with significant morbidity, including high anastomotic leakage rates in case of reconstruction and high wound complication rates with abdominoperineal resection.^9^ Complication rates of proctectomy range from 15% to 80%, with a 3%–9% mortality rate.^9^, ^10^, ^11^ Consequently, we tried non‐invasive methods first after the discussion with the patient.

A randomized controlled trial by Sahakitrungruang et al.^12^ showed that self‐administered rectal irrigation daily with oral antibiotics was more effective than 4% formalin therapy in patients with hemorrhagic CRP. In theory, rectal irrigation may reduce the fecal stream and bacterial load, thereby preventing superimposed infection. Moreover, this may allow the healing of rectal ulcers and ultimately, achieve hemostasis. Given the minimal risk for complications, rectal irrigation may be attempted before other invasive modalities.

We encountered a patient with severe hematochezia refractory to APC and diverting colostomy following high‐dose pelvic RT for cervical cancer. Treatment with rectal irrigation resulted in hemostasis after a few days. This study highlights the utility of rectal irrigation for controlling bleeding in patients with severe CRP refractory to other modalities. Before performing invasive procedures, rectal irrigation may be initially considered. Further well‐designed studies are needed to investigate the role of rectal irrigation in patients with severe CRP.

## CONFLICT OF INTEREST

The authors have no conflicts of interest to declare.

## AUTHOR CONTRIBUTIONS

All authors contributed significantly to the final manuscript. Sukit Pattarajierapan, Napapat Amornwichet, and Supakij Khomvilai were responsible for study conception, data acquisition, article drafting, and final approval prior to publication.

## ETHICAL APPROVAL

The study protocol was approved by the Institutional Review Board of Chulalongkorn University (IRB No. 713–64).

## CONSENT

The patient provided consent for the submission and publication of de‐identified case details and accompanying images.

## Data Availability

Data sharing is not applicable to this article.
